# Leptin receptor gene polymorphisms and morbid obesity in Mexican patients

**DOI:** 10.1186/s41065-016-0006-0

**Published:** 2016-02-22

**Authors:** Martin Edgardo Rojano-Rodriguez, Jose Luis Beristain-Hernandez, Beatriz Zavaleta-Villa, Pablo Maravilla, Mirza Romero-Valdovinos, Angelica Olivo-Diaz

**Affiliations:** 1 Departamento de Cirugia Endoscopica, Hospital General “Dr. Manuel Gea Gonzalez”, Mexico City, Mexico; 2 Departamento de Biologia Molecular e Histocompatibilidad, Hospital General “Dr. Manuel Gea Gonzalez”, SSA, Calzada de Tlalpan 4800, Col. Seccion XVI, 14080 Mexico City, Mexico; 3 Departamento de Ecologia de Agentes Patogenos, Hospital General “Dr. Manuel Gea Gonzalez”, Mexico City, Mexico

**Keywords:** Genetic susceptibility, *LEPR*, Morbid obesity, Single nucleotide polymorphisms

## Abstract

**Background:**

Human obesity is due to a complex interaction among environmental, behavioral, developmental and genetic factors, including the interaction of leptin (LEP) and leptin receptor (LEPR). Several *LEPR* mutations and polymorphisms have been described in patients with early onset severe obesity and hyperphagic eating behavior; however, some contradictory findings have also been reported. In the present study we explored the association of six *LEPR* gene polymorphisms in patients with morbid obesity.

**Findings:**

Twenty eight patients with morbid obesity and 56 non-obese Mexican Mestizo individuals were included. Typing of rs1137100, rs1137101, rs1805134, Ser492Thr, rs1805094 and rs1805096 *LEPR* polymorphisms was performed by PCR and allele specific hybridization. The *LEPR* Ser492Thr polymorphism was monomorphic with the presence of only the Ser492Thr-G allele. Allele C and genotype T/C for rs1805134 polymorphism were associated with susceptibility to morbid obesity (*p* = 0.02 and *p* = 0.03, respectively). No association was observed with any haplotype. Linkage disequilibrium (LD) showed that five polymorphisms (rs1137100, rs1137101, rs1805134, rs1805094 and rs1805096) were in absolute (D’ = 1) but none in perfect (r^2^ = 1) LD.

**Conclusions:**

Our results suggest that rs1805134 polymorphism could be involved in the development of morbid obesity, whilst none of the alleles of the *LEPR* gene, rs1137100, rs1137101, rs1805094 and rs1805096 were associated as risk factors. However, more studies are necessary to confirm or reject this hypothesis.

## Findings

Human obesity is due to a complex interaction among environmental, behavioral, developmental and genetic factors, the latter contributing to 40–70 % of the obese phenotype [1–3]. Leptin (LEP) is a hormone specifically produced by adipocytes, and its serum concentration is proportional to body fat mass which, in turn, has its amount regulated by the hypothalamic effects of LEP. Intravenous administration of LEP reduces appetite; while its deficiency increases food intake [4]. Its action occurs through the leptin receptor (LEPR), which is encoded by the *LEPR* gene. LEPR is a single-transmembrane-domain receptor of the cytokine-receptor family with widespread tissue distribution and several alternatively spliced isoforms [5].

Several *LEPR* mutations have been described in patients with early-onset of severe obesity and hyperphagic eating behavior [6, 7]. In contrast, a protective influence of two polymorphisms (rs1137100 and rs1137101) to higher blood pressure levels in men has been identified, increasing the protection when the carriers have the arginine allele in the two single nucleotide polymorphisms (SNPs) [8]. Thereby, several SNPs have been studied, and their replication in detail across different populations, for their potential association with obesity and its complication, has been stated. However, some contradictory findings have also been reported, adding the fact that there are scarce studies in non-Caucasian populations; therefore, the lack of data on this subject emphasizes the need for studies among and across different ethnic groups [9, 10]. In this work we explored the association of several *LEPR* gene polymorphisms with morbid obesity compared with non-obese Mexican Mestizo adults.

Blood samples were obtained from 28 patients with morbid obesity (mean age 39.6 ± 6.6 years, mean body mass index [BMI] 42.7 ± 6.5 Kg/m^2^) and 56 non-fat (mean age 32.7 ± 14.4, mean BMI 21.5 ± 1.5 Kg/m^2^) healthy unrelated volunteers. The sex of patients and controls was 89.7 and 93.3 % females, respectively. All participants were unrelated, with no consanguinity at all, and none couple was included. In addition to obesity, two patients presented hypothyroidism, one patient presented type 2 diabetes mellitus and arterial hypertension and one patient presented fat liver. This work complies with the current health laws of Mexico, and was approved by the Ethics and Research Committees of the Hospital General “Dr. Manuel Gea Gonzalez” with the reference number 04-92-2009. All participants were informed about the objectives of the study and were included only after providing written informed consent.

DNA was obtained from 10 ml of EDTA-peripheral blood using proteinase K and phenol/chloroform extraction [11]. The *LEPR* polymorphisms rs1137100 (Lys109Arg), rs1137101 (Gln223Arg), rs1805134 (Ser343Ser), Ser492Thr and rs1805096 (Pro1019Pro) were detected by PCR-restriction fragment length polymorphism (RFLP) technique, using primers described by Gotoda et al. [12] and Matsuoka et al. [13]. For allele determination of rs8179183 (Lys656Asn) SNP, a dot–blot format and the chemiluminescence method was used, employing the specific probes Lys656Asn-G: 5′-CTATGAAAAAGGAGAAAAATG-3' and Lys656Asn-C: 5'-CTATGAAAAACGAGAAAAATG-3′ ddUTP-Digoxigenin labelled [14, 15].

Allele frequencies (AF) and genotype frequencies (GF) were calculated by direct counting and were compared between patients and controls of each group. Chi-square analysis with Yate’s correction, considering *p* ≤ 0.05 as the minimum level of significance, was performed; exact Fisher test was used when appropriate. Relative risk was calculated as an odds ratio (OR). Ninety-five percent confidence intervals (95 % CI) were obtained by using Cornfield’s approximation. Haplotypes and linkage disequilibrium (LD) blocks were determined by confidence interval method using Haploview 4.2 software [16].

**Fig. 1:**
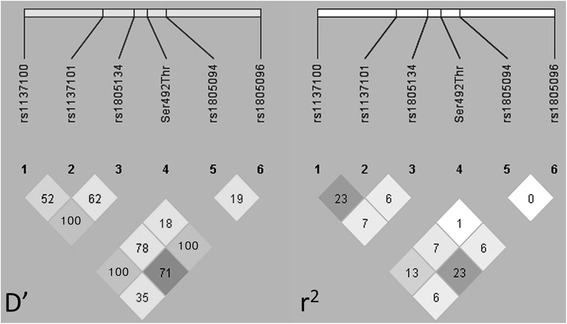
Linkage disequilibrium (LD) in *LEPR* polymorphisms between obese and control groups. The pairwise LD plot was created by Haploview 4.2. Within each *diamond* the pairwise standardized coefficient of LD (D’) or the correlation coefficient (r^2^) is presented in percentage. Standard color coding was used for the Haploview LD plots with the confidence bounds color scheme, for the D’ plot, numbers are representative LD and the logarithm of the odds are in color: *white* = strong evidence of recombination; *light grey* = uninformative; *dark grey* = strong evidence of LD; for r^2^ LD plots *white* (r^2^ = 0), *shades of grey* (0 < r^2^ < 1), *black* (r^2^ = 1). A marker pair is said to show moderate or usable LD if |D’| is between 0.33 and 0.5, and strong LD if |D’| is 0.5 or above (i.e. at least half the maximum value)

Table 1 summarizes the significant associations found between alleles, genotypes and haplotypes observed in patients with morbid obesity and in controls. Polymorphism Ser492Thr was monomorphic with the only presence of Ser492Thr-G allele. Allele C and genotype T/C for rs1805134 SNP were associated with susceptibility to morbid obesity (*p* = 0.02 and *p* = 0.03, respectively). Regarding the haplotype frequency, no association was observed; however, when shorter combinations were analyzed, haplotype rs1137101G-rs1805134C becomes associated with susceptibility (*p* = 0.036; OR [95 % CI] 3.4 [1.02–11.14]). Finally, according to LD plots generated in Haploview 4.2 (Fig. 1), the *LEPR* gene showed that five SNPs (rs1137100, rs1137101, rs1805134, rs1805094 and rs1805096) were in absolute but none in perfect LD.

**Table 1:** Alleles, genotypes and haplotypes frequencies of *LEPR* SNP*s* in a Co-dominant model

	Patients (*n* = 28)	Controls (*n* = 56)	*p* value^a^	OR (95 % CI)^b^
Alleles				
rs1137100-C (Lys109Arg)	0.45	0.43	0.99	1.08 (0.51–2.30)
rs1137100-T	0.55	0.57	0.99	0.92 (0.43–1.96)
rs1137101-G (Gln223Arg)	0.43	0.39	0.79	1.18 (0.58–2.39)
rs1137101-A	0.57	0.61	0.79	0.85 (0.41–1.72)
***rs1805134*** *-* ***C (Ser343Ser)*** ^c^	***0.17***	***0.06***	***0.02***	***3.41 (1.11–10.49)***
rs1805134-T	0.83	0.94	0.02	0.29 (0.09–0.90)
Ser492Thr-G	1	1	–	–
Ser492Thr-C	0	0	–	–
rs1805094-G (Lys656Asn)	0.22	0.12	0.13	2.02 (0.89–4.59)
rs1805094-C	0.78	0.88	0.13	0.49 (0.22–1.13)
rs1805096-G (Pro1019Pro)	0.40	0.40	0.93	0.97 (0.48–1.95)
rs1805096-A	0.60	0.60	0.93	1.03 (0.51–2.07)
Genotypes				
rs1137100 C/C	0.05	0.10	0.87	0.66 (0.07–6.01)
rs1137100 C/T	0.79	0.65	0.42	1.85 (0.19–0.59)
rs1137100 T/T	0.16	0.25	0.65	0.64 (0.16–2.57)
rs1137101 A/A	0.32	0.30	0.88	1.09 (0.38–3.16)
rs1137101 A/G	0.50	0.61	0.54	0.65 (0.24–1.76)
rs1137101 G/G	0.18	0.09	0.45	2.28 (0.55–9.43)
***rs1805134 T/T*** ^d^	***0.65***	***0.89***	***0.03***	***0.24 (0.07–0.83)***
***rs1805134 T/C*** ^d^	***0.35***	***0.11***	***0.03***	***4.01 (1.20–13.41)***
Ser492Thr G/G	1.00	1.00	–	–
rs1805094 C/C	0.59	0.77	0.13	0.43 (0.17–1.13)
rs1805094 C/G	0.38	0.22	0.17	2.19 (0.83–5.77)
rs1805094 G/G	0.03	0.01	0.82	2.09 (0.13–34.61)
rs1805096 A/A	0.33	0.33	0.84	1.05 (0.37–2.92)
rs1805096 A/G	0.54	0.54	0.82	1.01 (0.38–2.66)
rs1805096 G/G	0.13	0.13	0.80	0.99 (0.23–4.20)
Haplotypes^e^				
CGTGCA	0.266	0.271	0.94	0.97 (0.47–2.01)
TATGCG	0.173	0.223	0.47	0.74 (0.32–1.69)
TATGCA	0.108	0.106	0.98	1.01 (0.36–2.88)
CATGCG	0.078	0.117	0.44	0.64 (0.20–2.00)
TATGGA	0.084	0.088	0.92	0.94 (0.30–3.01)
TGCGCA	0.073	0.058	0.70	1.28 (0.36–4.62)
CATGCA	0.037	0.041	0.91	0.91 (0.17–4.92)
TGTGCG	0.037	0.029	0.79	1.27 (0.22–7.53)
TATGGG	0.047	0.024	0.39	2.12 (0.37–12.03)
TACGGG	0.032	0.005	0.13	7.97 (0.34–185.4)
TGTGCA	0.005	0.016	0.56	0.34 (0.01–15.99)

^*^ *p* value with Yates correction; ^b^Odds ratio (95 % confidence interval); ^c^Statistical power = 0.632; ^d^Statistical power = 0.679; ^e^ *LEPR* haplotypes: rs1137100-rs1137101-rs1805134-Ser492Thr-rs1805094-rs1805096. Characters in bold and italics indicate statistically significant values

There are data pointing to the fact that the Hispanic population exhibits predisposition towards obesity ([17, 18], http://www.who.int/topics/obesity/en/). Therefore, it is important to study the genetic mechanisms that predispose individuals to this pathology. In the present study, of the six SNPs analyzed, only allele C and genotype T/C for rs1805134 (Ser343Ser) SNP was associated with morbid obesity. Interestingly, this polymorphism has been scanty studied in obesity; when association between serum lipids and *LEP*/*LEPR* gene polymorphisms in obese Japanese children was studied, the rs1805134 SNPs showed a significant relationship with serum lipid profile, since lower triglyceride levels were obtained in rs1805134 C/C homozygotes [19]. In another study performed in Spanish adults with obesity, no significant case–control differences were found in allele/genotype frequencies for rs1137100, rs1137101, rs1805134 and rs8179183 SNPs [20]. Since rs1805134 SNP addresses a synonymous amino acid change and, according to our results, it was in absolute LD with both flanking SNPs (rs1137100 and rs1805096) in the *LEPR* gene, it suggests that SNPs have not been affected by recombination events, which is in concordance with the literature. The present observations allow us to speculate about the presence of others polymorphisms, probably located in introns, close to rs1805134 SNP in absolute and perfect LD, which might regulate the expression of exon 9 of *LEPR* gene, as it has been described for other Eukaryotes genes [21].

Regarding rs1137100, rs1137101 and rs8179183 SNPs, our data are similar with those reported in others Mexican populations; Guizar-Mendoza et al. [22] assessed rs1137101 and rs1805096 LEPR polymorphisms in Mexican adolescents from Guanajuato state, finding no differences in the genotype frequencies of these SNPs between obese and non-obese participants. Furthermore, another study in Mexican children and adolescents from Colima state, analyzed rs1137100, rs1137101 and rs8179183 SNPs, and no statistically significant association with obesity was found in any of the alleles [18].

The main limitation of the present study was the small sample size; nevertheless we were able to demonstrate statistically significant differences with an adequate power between case and control groups. Rosmond [10] has made harsh criticisms on the association with risk factors of case–control genetic studies, highlighting that the current literature linking central obesity to genetic variants has many reports of associations that either cannot be reproduced or corroborated. The present study, as opposed to Rosmond’s criticism, does replicate the findings reported by other researchers in the same ethnic group, supporting the consistency of allele and genotype frequency for some *LEPR* polymorphisms in a specific group of participants; therefore, we propose that the rs1805134 SNP is interesting and might be involved in morbid obesity; however, more studies are necessary to confirm or reject this hypothesis.

## Abbreviations

LEP: leptin
LEPR: leptin receptor
SNP: single nucleotide polymorphism
BMI: body mass index
DNA: deoxyribonucleic acid
EDTA: ethylenediaminetetraacetic acid
PCR: polymerase chain reaction
RFLP: restriction fragment length polymorphism
AF: allele frequency
GF: genotype frequency
LD: linkage disequilibrium
OR: odds ratio
CI: confidence interval
